# Evaluation of a combinatorial approach to prion inactivation using an oxidizing agent, SDS, and proteinase K

**DOI:** 10.1186/1746-6148-9-151

**Published:** 2013-07-25

**Authors:** Jodi D Smith, Eric M Nicholson, Justin J Greenlee

**Affiliations:** 1 Virus and Prion Research Unit, National Animal Disease Center, USDA, Agricultural Research Service, 1920 Dayton Ave, Ames, IA 50010, USA; 2 Department of Veterinary Pathology, College of Veterinary Medicine, Iowa State University, Ames, IA 50011, USA

**Keywords:** Inactivation, Prion, Proteinase, Scrapie, Sodium dodecyl sulfate, Sodium percarbonate

## Abstract

**Background:**

Prions demonstrate an unusual resistance to methods effective at inactivating conventional microorganisms. This has resulted in a very tangible and difficult infection control challenge to the medical and veterinary communities, as well as animal agriculture and related industries. Currently accepted practices of harsh chemical treatments such as prolonged exposure to sodium hydroxide or sodium hypochlorite, or autoclaving are not suitable in many situations. Less caustic and more readily applicable treatments to contaminated environments are therefore desirable. We recently demonstrated that exposure of the RML scrapie agent to a commercial product containing sodium percarbonate (SPC-P) with or without sodium dodecyl sulfate (SDS) rendered PrP^Sc^ sensitive to proteinase K (PK), but did not eliminate infectivity. The current study was designed to evaluate the efficacy of a combinatorial approach to inactivating prions by exposing RML-positive brain homogenate to SPC-P and SDS followed by PK. Treated samples were evaluated for PrP^Sc^-immunoreactivity by western blot, and residual infectivity by mouse bioassay.

**Results:**

Treatment of infected brain homogenate with SPC-P and SDS followed by PK exposure resulted in a 4–5 log_10_ reduction in infectivity when bioassayed in *tg*a*20* mice.

**Conclusions:**

This study demonstrates that exposure of the RML scrapie agent to SPC-P and SDS followed by PK markedly reduces, but does not eliminate infectivity. The results of this study encourage further investigation into whether consecutive or concomitant exposure to sodium percarbonate, SDS, and a protease may serve as a viable and non-caustic option for prion inactivation.

## Background

Prions are the causative agent of the transmissible spongiform encephalopathies (TSE) and consist predominantly, if not solely, of an abnormally folded, partially protease resistant isoform of the cellular prion protein (termed PrP^Sc^) [1]. However, the exact nature of the infectious agent remains open to debate with the identification of protease sensitive forms of disease-associated PrP [2-4] and retention of infectivity after complete PrP digestion [5]. Regardless of their specific identity, prions are notoriously difficult to inactivate, withstanding methods effective against conventional pathogens, such as moderate heating, ultraviolet irradiation, and formalin exposure [6]. Prions are transmitted more efficiently within a species, but interspecies transmission is also possible – the most infamous example being transmission of bovine spongiform encephalopathy (BSE) to humans resulting in the variant form of Creutzfeldt-Jakob disease (vCJD) [7-9]. Because BSE poses a lethal zoonotic disease risk, effective prion decontamination methods that can be applied on a large scale, such as in abattoirs, are desirable to further minimize the risk of zoonotic transmission. Efficacious decontamination procedures that can be applied to environmental settings are also desirable to aid in the control of other TSEs of veterinary and public health importance such as scrapie and chronic wasting disease, which are horizontally transmitted [10,11]. Ideally, effective methods would be non-hazardous to personnel applying them on a large scale and reasonably sensitive to ecological systems when applied to contaminated outdoor environments.

Current decontamination recommendations by the World Health Organization, depending on material to be sterilized, include autoclaving at 134°C for up to 1 hour, or prolonged exposure to 1 N sodium hydroxide or ≥ 20,000 ppm sodium hypochlorite [12]. The most recent edition of the US Department of Health and Human Service’s *Biosafety in Microbiological and Biomedical Laboratories* also recognizes the phenolic disinfectant Environ LpH (Steris Corp.) as an acceptable decontaminating solution for surfaces and reusable instruments [13]. Inactivation treatments with sodium hydroxide or sodium hypochlorite are especially detrimental to delicate surgical and diagnostic equipment, spurring research into less caustic alternatives. Recent lines of investigation have included treatment of contaminated material with sodium dodecyl sulfate (SDS), proteolytic enzymes, or peroxygen compounds with variable success. Sodium dodecyl sulfate has long been known to affect prion infectivity, but its effectiveness varies with the prion strain to which it is applied. Prior investigations have demonstrated minimal effects on CJD infectivity [14], but up to a 3 log_10_ reduction on scrapie infectivity [15]. More recently, SDS in combination with NaOH has been shown to successfully inactivate the 263 K hamster scrapie agent, resulting in a > 5.5 log_10_ reduction in infectivity [16,17]. Proteases have also been reported to have prion inactivation potential [18-22]. Broad spectrum proteases, such as proteinase K (PK) [18] and pronase [19], have been shown to substantially reduce infectivity after prolonged exposure times. More recently, a genetically engineered variant of subtilisin applied under alkaline conditions was shown to reduce infectivity of a mouse-adapted BSE strain by > 7 logs [21]. While these reagents have demonstrated anti-prion activity on their own, combining proteases and SDS appears to enhance their prion inactivating abilities. In two independent studies, pronase [19] or pronase and PK [23] in combination with SDS resulted in greater reductions in infectivity versus protease(s) alone. Peroxygens, such as liquid hydrogen peroxide [24-26] and peracetic acid [27], have been shown to promote limited inactivation. However, more recent studies have demonstrated significant reductions in infectivity for hamster-adapted scrapie and mouse-adapted BSE strains using vaporized hydrogen peroxide [25,28].

Sodium percarbonate is an oxidizing agent composed of sodium carbonate and hydrogen peroxide (2 Na_2_CO_3_ • 3 H_2_O_2_). It has a high degree of environmental compatibility, with degradation products consisting of water, oxygen, and sodium carbonate, and generates a pH of 10–11 in aqueous solution. Until recently, its efficacy with regard to prion inactivation had not been reported. In a prior study [29], we found that exposure of RML-positive brain homogenate to a sodium percarbonate-containing product (SPC-P) with or without SDS was unsuccessful at eliminating infectivity, but did increase sensitivity of PrP^Sc^ to PK digestion. The current study builds upon those results, examining a combinatorial approach to inactivating prions by exposing infected material to SPC-P and SDS followed by treatment with PK. Brain homogenate from terminally ill C57BL/6 mice positive for the mouse-adapted RML strain of scrapie was treated with a combination of SPC-P and 2.5% w/v SDS for 30 minutes at room temperature followed by exposure to PK. Western blot (WB) analysis was used to detect residual PrP^Sc^ in treated samples, and residual infectivity was assayed by intracranial inoculation into prion protein overexpressing *tg*a*20* mice [30].

## Results

### Immunoblotting

**Figure 1:**
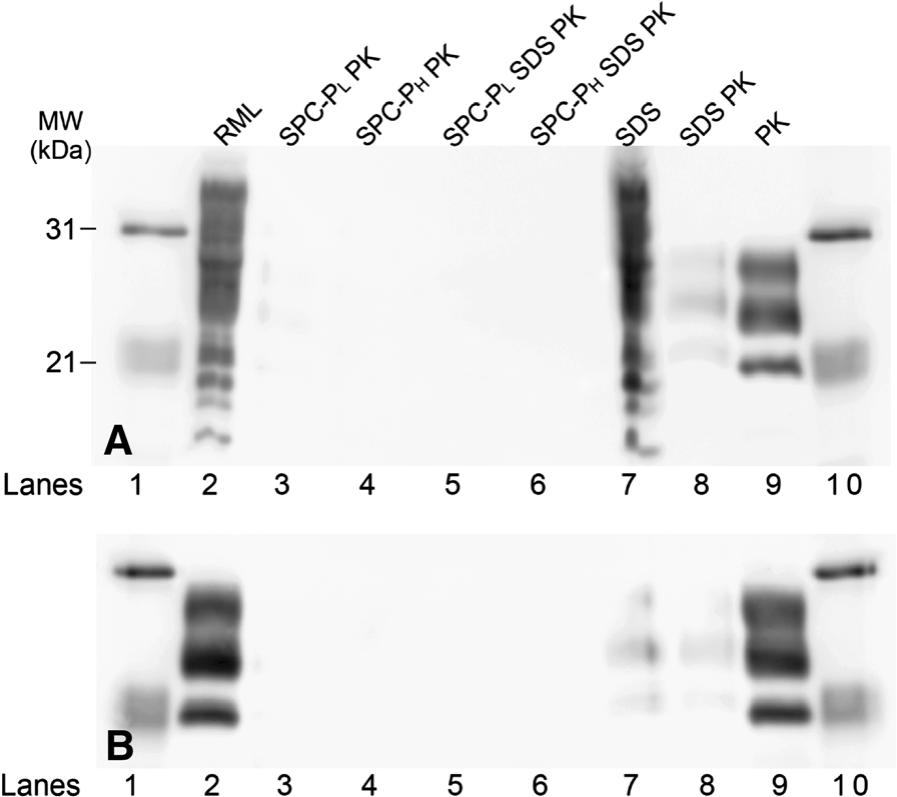
**Western blot of PrP in treated brain homogenate from RML scrapie-affected C57Bl/6 mice without (A) or with (B) PK pretreatment prior to immunoblotting.** PrP-immunoreactivity was faintly detectable in samples exposed to SPC-P_L_ alone followed by PK (lane 3) and undetectable in samples treated with either SPC-P_H_ alone or with SPC-P_L_ or SPC-P_H_ combined with SDS followed by PK (lanes 4–6). Control samples included untreated RML brain homogenate (lane 2), SDS treatment only (lane 7), SDS followed by PK (lane 8), and PK treatment only (lane 9). Additional exposure of samples to PK prior to immunoblotting (as part of the WB protocol for PrP^Sc^) did not result in loss of PrP^Sc^ immunoreactivity in control samples. Lanes 1 and 10, molecular weight marker. Abbreviations: MW, molecular weight.

Residual PrP^Sc^ in brain homogenate treated with a low or high concentration of the SPC-containing product (SPC-P_L_ and SPC-P_H_, respectively) in combination with 2.5% w/v SDS followed by exposure to PK was assayed via western blot (WB). Immunoblots were performed in triplicate with representative blots presented in Figure 1. Immunoreactivity for the di-, mono-, and unglycosylated forms of PrP^Sc^ was faintly detectable in samples exposed to SPC-P_L_ alone followed by PK (Figure 1, lane 3). Immunoreactivity was undetectable in samples treated with SPC-P_H_ alone followed by PK exposure, or either concentration of SPC-P combined with SDS and followed by PK (Figure 1, lanes 4–6). Control samples exposed to SDS alone, PK alone, or SDS followed by PK retained PrP-immunoreactivity (Figure 1A and B, lanes 7–9).

### Mouse bioassay

Residual infectivity in treated samples was assayed via intracranial inoculation of *tg*a*20* mice. The average number of days to terminal disease for the positive control group (untreated RML-positive brain homogenate) was 65.5 ± 3.4 days, which is typical of RML disease kinetics in *tg*a*20* mice inoculated intracranially [30]. As previously reported [29], ten-fold serial dilutions of this stock resulted in increased mean incubation times and survival percentages (Table 1). The survival curves generated from these data were used as the comparative standard when evaluating survival time in mice inoculated with treated samples and for approximating reductions in infectivity. Consistent clinical signs observed in scrapie-affected mice included ataxia that progressed to a listing or rolling gait in some cases, pelvic limb paresis, and lethargy. All but 2 animals in the negative control group (treated RML-negative brain homogenate) survived until study termination. One mouse was euthanized due to severe hydrocephalus and the cause of death of the other mouse was undetermined.

**Table 1:** **Average incubation times and survival percentages of *tg*a*20 *mice inoculated intracranially with serially diluted or treated RML scrapie brain homogenate**

**Group**	**Dilution or treatment**	**Mean incubation time in days ± SD (% survival)**
	RML titration series^*^	
	10^0^	66 ± 3.4 (0%)
	10^-1^	74 ± 3.3 (0%)
	10^-2^	83 ± 6.1 (0%)
	10^-3^	128 ± 29.1 (22%)
	10^-4^	184 ± 39.6 (78%)
	10^-5^ through 10^-12^	N/A (100%) – study termination
	*Treated RML*	
	SPC-P_L_^*^	67 ± 2.6 (0%)
	SPC-P_H_^*^	70 ± 6.0 (0%)
	SPC-P_L_ + SDS^*^	93 ± 9.7 (0%)
	SPC-P_H_ + SDS^*^	93 ± 7.6 (0%)
1	SPC-P_L_ ∏ PK	107 ± 39.0 (11%)
2	SPC-P_H_ ∏ PK	98 ± 16.8 (0%)
3	SPC-P_L_ + SDS ∏ PK	431 ± 72.4 (73%)
4	SPC-P_H_ + SDS ∏ PK	108^^^ (88%)
5	SDS control	85 ± 12.3 (0%)
6	PK control	62 ± 5.2 (0%)
7	SDS ∏ PK control	86 ± 7.1 (0%)

*Smith *et al.*[29]; ^only 1 mouse in this group was euthanized due to clinical scrapie.

*SD* standard deviation.

**Figure 2:**
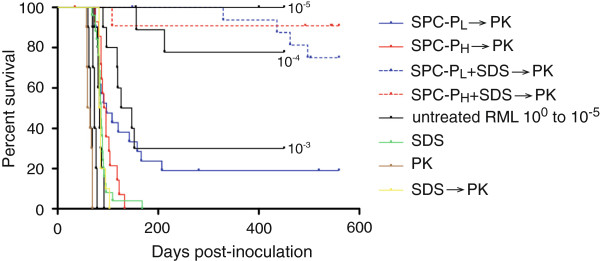
**Effect of various treatment conditions on scrapie infectivity in *tg*a*20 *mice.** Kaplan-Meier survival curves were generated to compare treatment conditions to 10-fold serial dilutions of RML scrapie in *tg*a*20* mice. Treatment with PK alone had no effect on infectivity. Treatment with SDS alone or SDS followed by PK (SDS → PK) yielded an approximate 2 log_10_ reduction in infectivity with 0% survival. Similarly, treatment with either SPC-P_L_ or SPC-P_H_ alone followed by exposure to PK resulted in a 2–3 log_10_ reduction in infectivity. The combination of SPC-P_L_ or SPC-P_H_ with SDS followed by PK resulted in a 4–5 log_10_ reduction in infectivity with 73% and 88% survival, respectively. Abbreviations: SPC-P_L_ or _H_, low or high concentration sodium percarbonate-based product; SDS, sodium dodecyl sulfate; PK, proteinase K.

Mice inoculated with samples exposed to SPC-P_L_ or SDS-P_H_ alone followed by PK had mean incubation times of 107 ± 39 and 98 ± 16.8 days, respectively, corresponding to an approximate 2–3 log_10_ reduction in infectivity (Figure 2). Two of 19 mice (11%) in the SPC-P_L_ group survived until study termination, while all mice in the SDS-P_H_ group were euthanized due to severe clinical signs of scrapie. Exposure to the combination of SPC-P and SDS followed by PK resulted in a 4–5 log_10_ reduction in infectivity (Figure 2). The mean incubation time for this combination using SPC-P_L_ was 431 ± 72.4 days, with 73% of mice surviving until study termination. Two mice from this group were censored from survival statistics due to intercurrent disease. For the combination using SPC-P_H_, only 1 mouse developed clinical signs at 108 days post-inoculation and was definitively diagnosed with scrapie (88% survival). Four mice in this group died within 7 days of inoculation due to complications from the procedure, and 4 additional mice were later censored due to intercurrent disease. Mice inoculated with samples exposed to PK alone had an average incubation time of 62 ± 5.2 days with 0% survival. Mice inoculated with samples exposed to SDS alone followed by PK had a mean incubation time of 86 ± 7.1 days, corresponding to an approximate 2 log_10_ reduction in infectivity but 0% survival.

## Discussion

Oxidizing agents have been used with variable success in prion inactivation studies. Recent studies applying vaporized hydrogen peroxide (VHP) to prion-contaminated stainless steel surfaces have demonstrated significant reductions in infectivity for hamster-adapted scrapie and mouse-adapted BSE strains [25,28]. A 4.5-5.6 log_10_ reduction in infectivity of the hamster-adapted 263 K scrapie agent was demonstrated after treatment of contaminated steel wires with VHP alone or in combination with an enzymatic cleaner [28]. Similarly, exposure of wires contaminated with the mouse-adapted 6 PB1 strain of BSE to VHP alone resulted in a reduction of > 5.5 logs [25]. In contrast, a ≤ 1 log_10_ reduction in infectivity was demonstrated with liquid hydrogen peroxide [25]. Our prior work examining SPC-P treatment alone also demonstrated little to no (≤ 1 log_10_) reduction in infectivity of the RML scrapie agent [29]. While VHP has demonstrated greater efficacy against prions, we felt the efficacy of liquid hydrogen peroxide might be improved in combination with other protein-disrupting conditions and compounds, such as the alkaline conditions attained with SPC solution and the denaturing and proteolytic effects of SDS and PK, respectively.

In this report, we investigated the viability of combining sodium percarbonate, SDS, and PK to inactivate the RML scrapie agent. Treated samples were evaluated for PrP^Sc^ immunoreactivity by WB and residual infectivity by mouse bioassay. Product choice and treatment conditions for this study were defined based on our aforementioned study in which we demonstrated exposure of the RML scrapie agent to SPC-P with or without SDS increased the sensitivity of PrP^Sc^ to PK, but did not eliminate infectivity. The results of the current study demonstrating loss of PrP^Sc^ immunoreactivity after treatment with SPC-P_H_ alone, or either concentration of SPC-P combined with SDS, followed by PK are in agreement with previous findings. Exposure of brain homogenate to this combination resulted in a > 4 log_10_ reduction in infectivity, with 73% of challenged mice from the SPC-P_L_ + SDS → PK group and 88% of mice from the SPC-P_H_ + SDS → PK group surviving until study termination. Treatment with PK alone had no effect on infectivity, while treatment with SDS alone or SDS → PK resulted in an approximate 2 log_10_ reduction, but 0% survival. Treatment with either SPC-P_L_ or SPC-P_H_ alone followed by PK also had a modest impact on infectivity resulting in a 2–3 log_10_ reduction in infectivity, but with 11% and 0% survival, respectively. In our previous study we reported that SPC-P_L_ or SPC-P_H_ alone had little to no effect on infectivity (≤ 1 log_10_), but in combination with SDS resulted in a 2–3 log_10_ reduction in infectivity but with few survivors. Taken together, these data indicate the most viable approach is utilizing all components in combination.

Both concentrations of SPC-P used in this study generated a pH of approximately 11 in solution. We previously evaluated the effect of pH on this particular inoculum and demonstrated loss of PrP^Sc^ immunoreactivity after incubation of brain samples buffered with 0.35 M Na_2_HPO_4_ (pH 11.0). It is well established that prion infectivity is reduced under extremely basic conditions, such as exposure to NaOH (pH 12–14) [31-33]. While the pH generated by SPC-P is lower at near 11, it appears to be a favorable characteristic of the compound with regard to PrP^Sc^ protease sensitivity and is likely contributing to the mechanism of infectivity reduction observed in this study. Also, although treatment with this combination rendered PrP^Sc^ sensitive to PK and substantially decreased infectivity, it did not completely eliminate infectivity. This was not unexpected, as a number of studies have demonstrated dissociation of PrP^Sc^ and infectivity [2,5,34,35]. Our results support the conclusion that biochemical analysis alone is insufficient for determination of prion infectivity. The observed PrP^Sc^/infectivity mismatch warrants a number of considerations including WB sensitivity, epitope disruption by inactivation treatments, and alternative infectious agents to solely PrP^Sc^. It is possible the amount of residual PrP^Sc^ in our treated samples was below the detection limit of our WB, or it may be that a true dissociation of PrP^Sc^ and TSE infectivity exists supporting the actuality of alternative infectious agents to PrP^Sc^[36]. A recent study has demonstrated poor correlation between infectivity and WB results for sheep scrapie and sheep BSE [35], in line with observations that PK-sensitive PrP particles are associated with disease [2,37].

## Conclusions

This study is the first to report the efficacy of a novel combination of oxidizing agent, detergent, and protease to inactivate prions. Exposure of the RML scrapie agent to an SPC-containing product combined with SDS followed by PK exposure substantially reduced prion infectivity by 4–5 logs. While this combination did not completely eliminate infectivity, it is feasible that further investigation and protocol modification may result in improved efficacy yielding a non-hazardous and widely applicable solution for prion decontamination.

## Methods

### Inactivation of inoculum

Brain samples from negative control or RML-positive mice were prepared as 10% w/v brain homogenates in phosphate buffered saline as described previously [29]. Homogenates were diluted to a concentration of 5% in either a 2.1% (SPC-P_L_, pH 10.7) or 21.0% (SPC-P_H_, pH 10.6) solution of a commercial product containing SPC (OxiMagic™, Clorox Company; 50-60% SPC) with or without 2.5% sodium dodecyl sulfate (SDS). Manufacturer’s instructions on concentration were followed (2.1% working solution), but additional parameters were experimentally defined. Samples were agitated under ambient oxygen in microcentrifuge tubes at 25°C for 30 min. Next, proteinase K (PK) (USB Corporation) was added to a final concentration of 0.08 mg/mL and samples were incubated at 48°C for 40 min. Pefabloc (Roche) was added to a final concentration of 1 mg/mL to quench PK activity. Samples were then diluted with sterile saline to a final concentration of 1% for inoculation (final pH values of 10.3 and 10.6 for SPC-P_L_ and SPC-P_H_-treated samples, respectively). Samples were inoculated intracranially (see below) into 10–20 *tg*a*20* mice per treatment condition. Mice (n = 10/group) inoculated with RML-positive brain homogenate exposed to 0.08 mg/mL PK alone at 48°C for 40 min, or 2.5% w/v SDS alone or followed by PK (as above) were included as PK-only, SDS-only, and SDS PK controls, respectively. Mice (n = 10/group) inoculated with RML-negative brain homogenate treated with SPC-P with or without SDS served as negative controls. Samples were stored at −20°C until thawing for western blot analysis and inoculation into *tg*a*20* mice. Mice were inoculated (see below) within 12–24 hours of sample treatment.

### Western blotting

Treated samples from each inactivation condition along with controls were examined for PrP^Sc^ via western blot. Pretreatment of brain homogenate with PK was performed on one set of blots and omitted on repeated blotting of the same samples. Briefly, samples were digested with PK using a final concentration of 0.08 mg/mL of at 48°C for 40 min. Pefabloc was added to a final concentration of 0.1 mg/mL to quench PK activity. Samples were dissolved in SDS-PAGE sample buffer and analyzed by standard western blotting procedures. A tissue equivalent of 1.0 mg was loaded onto the gel for each sample. PrP^Sc^ was detected using monoclonal antibody 6H4 (Prionics, La Vista, NE) at a 1:10,000 (0.1 μg/mL) dilution applied for 1 hr at room temperature or 4°C overnight. A biotinylated sheep anti-mouse secondary antibody and a streptavidin-horseradish peroxidase (HRP) conjugate (GE Healthcare, Pittsburgh, PA) were used in conjunction with the ECL Plus detection kit (GE Healthcare) to detect immunolabeling. Secondary antibody and streptavidin-HRP conjugate incubations were conducted at room temperature for 1 hour. Blots were developed using the Typhoon 9410 Variable Mode Imager (Molecular Dynamics, Sunnyvale, CA).

### Mouse bioassay

Comparative survival curves were generated as previously described [29] in mice of the B6;129S7-Prnp^tm1Cwe^Tg(Prnp)a20Cwe/CweCnrm (*tg*a*20*) line [30]. For all inoculations, *tg*a*20* mice were anesthetized with isoflurane and a 30 gauge tuberculin syringe was used to inject 20 microliters of brain homogenate into the right cerebral hemisphere at a depth of 3–5 mm. Mice were monitored for 48 hours post-inoculation for procedure-related adverse events. Mice were then monitored daily and euthanized when they displayed unequivocal neurological signs, or at the time of study termination (560 days post-inoculation). Scrapie diagnosis was confirmed with a combination of WB, histopathology, and immunohistochemistry for PrP. Experimental and control groups included mice inoculated with RML brain homogenate treated as follows: 1) SPC-P_L_ followed by PK, 2) SPC-P_H_ followed by PK, 3) SPC-P_L_ + SDS followed by PK, 4) SPC-P_H_ + SDS followed by PK, 5) SDS only at 25°C x 30 min, 6) PK only at 48°C for 40 min, 7) SDS followed by PK (Table 1). This experiment was carried out in accordance with the Guide for the Care and Use of Laboratory Animals (Institute of Laboratory Animal Resources, National Academy of Sciences, Washington, DC) and was approved by the National Animal Disease Center’s Animal Care and Use Committee (protocol #2422).

### Statistics

Kaplan-Meier survival curves were generated using statistical software (Prism version 4.0, GraphPad Software). To estimate reductions in infectivity, survival curves from treated groups were compared to those of the titration study using the logrank test with a level of statistical significance of 0.05. Mice that died within the first 21 days PI due to complications related to intracranial inoculation or were removed from the study for humane reasons (e.g. intercurrent disease, injuries) prior to developing clinical signs were censored and not included in survival analyses.

## Abbreviations

BSE: Bovine spongiform encephalopathy; CJD: Creutzfeldt-Jakob disease; MW: Molecular weight; PI: Post-inoculation; PK: Proteinase K; PrPSc: Disease-associated isoform of the prion protein; RML: Rocky Mountain Laboratory strain of the scrapie agent; SDS: Sodium dodecyl sulfate; SPC-P: Sodium percarbonate-based product; SPC-PL: Low concentration SPC-P; SPC-PH: High concentration SPC-P; TSE: Transmissible spongiform encephalopathy; WB: Western blot.

## Competing interests

The authors declare that they have no competing interests.

## Authors’ contributions

JDS conceived of the study, carried out the western blot and animal bioassay studies, and drafted the manuscript. EMN participated in the design of the study and interpretation of results. JJG participated in the design of the study and helped to draft the manuscript. All authors read and approved the final manuscript.

## Authors’ information

JDS and JJG are Research Veterinary Medical Officers in the NADC VPRU. JDS is a Postdoctoral Research Associate. EMN is a Research Chemist and Lead Scientist in the VPRU.
